# Nonpharmaceutical therapy for autism spectrum disorder: A protocol for systematic review and network meta-analysis

**DOI:** 10.1097/MD.0000000000028811

**Published:** 2022-02-18

**Authors:** Xin Jiang, Min Song, Weixun Qin, Jiang Xiao, Xiaoqing Xu, Qing Yuan

**Affiliations:** a Medical College of Acu-Moxi and Rehabilitation, Guangzhou University of Chinese Medicine, Guangzhou, China; b The Fourth Department of Acupuncture and Moxibustion, Shaanxi Traditional Chinese Medicine Hospital, Xi’an, China.

**Keywords:** autism, network meta-analysis, nonpharmacological therapies, protocol

## Abstract

**Background:**

Autism spectrum disorder (ASD) is a widespread developmental disorder of the nervous system with an unclear etiology and pathogenesis. Its global incidence is currently increasing, and no effective drugs are available to improve its core symptoms. Nonpharmaceutical therapy can effectively relieve the core symptoms of autism, has fewer side effects than drugs, and is easily accepted by patients. This systematic and network meta-analysis aimed to evaluate the impact of non-pharmaceutical therapy on autism to explore preferable therapeutic options for autism.

**Methods:**

Online databases, including the China National Knowledge Infrastructure [CNKI], SinoMed, Wanfang Database [WF], China Science and Technology Journal Database [VIP], MEDLINE, Web of Science, EMBASE, and the Cochrane Library will be searched for randomized controlled trials of nonpharmacological interventions for autism published before October 2021. Two researchers will be independently responsible for the literature screening, data extraction, and quality assessment. Standard paired and Bayesian network meta-analyses will be performed using RevMan 5.3 Software and GEMTC 0.14.3, to compare the efficacy and safety of different nonpharmacological regimens.

**Results:**

The results of this systematic and network meta-analysis will be submitted to a peer-reviewed journal for publication.

**Conclusion:**

This study provides a comprehensive and reliable evidence-based reference for the efficacy and safety of different non-pharmacological interventions for autism.

**PROSPERO registration number:**

CRD 42021275571

## 1 Introduction

Autism spectrum disorder (ASD) is a serious developmental disorder of the nervous system with core symptoms of social dysfunction, communication barriers, narrow interests, and rigid behaviors.^[1]^ This disease seriously threatens children's physical and mental health and greatly impacts their family life quality.^[2]^ Although its etiology and pathogenesis remain unclear, this illness is mainly associated with genetic,^[3]^ perinatal,^[4]^ environmental,^[5]^ and immune factors.^[6]^ Most researchers believe that ASD is caused by the joint action of genes and environmental factors.^[7]^ Its global incidence is approximately 1% to 2%,^[8]^ and its impact on men is 4 to 5 times higher than that on women.^[9]^ Over the past few decades, the incidence of ASD has continuously increased,^[^10^,^^11^^]^ causing a huge economic burden on society and attracting the attention of the international community. The prevalence of ASD among 8-year-old children in the United States increased from 6.7% in 2000 to 14.6% in 2012,^[12]^ and the annual expense was as high as 236 billion dollars.^[13]^

The current treatment for autism is mainly behavioral interventions combined with drug therapy.^[14]^ Behavioral intervention can improve language, cognitive abilities, adaptive behavior, and social skills, and reduce anxiety and aggression; however, the required cooperation among families, communities, and schools costs a great quantity of manpower and material resources, and the effect is not stable.^[^15^–^18^]^ Medications such as antidepressants, stimulants, antipsychotics, alpha agonists, and anticonvulsants are often used to relieve anxiety, ADHD symptoms, compulsions, and other repetitive behaviors, mood lability, irritability, aggression, and sleep disturbance. However, these drugs cannot assuage the core symptoms of communication skills and stereotyped behaviors and can induce multiple side effects such as sedation, increased appetite and weight gain, disrupted sleep, prolactin elevation, and extrapyramidal symptoms.^[^19^–^21^]^

Non-pharmacological treatments, such as yoga, acupuncture, massage, music therapy, and manual therapy, are simple and economical methods that can effectively alleviate the core symptoms of ASD, such as social barriers, communication barriers, and stereotyped behaviors.^[^22^–^26^]^ Given the lack of reported adverse reactions or interactions associated with non-pharmacological therapies, this treatment is more easily accepted by patients than prescription drugs. However, current meta-analyses have only compared the effectiveness of a single non-drug therapy. A network meta-analysis is necessary to evaluate the impact of different types of non-drug treatment on the core symptoms of patients with ASD and to explore the best non-drug options.

This systematic review and network meta-analysis aimed to evaluate the potential safety and effectiveness of different types of nonpharmacological interventions for ASD.

## 2 Methods

### 2.1 Objectives and registration

This systematic review and network meta-analysis aimed to evaluate the efficacy and safety of different nonpharmaceutical interventions for ASD.

This study will be reported with the Preferred Reporting Items for Systematic Review and Meta-Analyses statement.^[27]^ The systematic review and network meta-analysis protocol was registered in the International Prospective Register of Systematic Reviews (registration number: CRD 42021275571).

### 2.2 Eligibility criteria

#### 2.2.1 Types of studies

Regardless of the language or publication status, only clinical randomized controlled trials (RCTs) of nonpharmacological treatment for children with autism will be included. Repeat published studies, non-RCTs, and laboratory studies were excluded.

#### 2.2.2 Types of participants

Participants were adolescents between the ages of 3 and 17 years who met the diagnostic criteria for ASD in the Diagnostic Statistical Manual or the International Classification of Diseases, regardless of sex, race, study area, or educational status.

#### 2.2.3 Types of interventions

Interventions included any type of clinically performed non-pharmaceutical therapy for patients with autism, such as music therapy, play therapy, applied behavior analysis, TEACCH training, relationship development intervention, manual therapy, massage, acupuncture, and yoga.

**Control:** no invention or treatment other than non-pharmaceutical therapy (pharmacological treatment, placebo, and usual care).

#### 2.2.4 Types of outcome measures

Primary outcomes included changes in the core syndromes (social disorder, stereotyped behavior, narrow interests, and language barriers) of autism. Evaluation will be performed using the symptom scores of the Childhood Autism Rating Scale (CARS) and the Autism Rating Scale (ABC). The CARS is one of the most widely used autism rating scales that is suitable for children over 2 years of age, has good reliability and validity, and can distinguish autism from retardation and judge its severity. ABS has 57 items of behavioral characteristics in 5 aspects, including sensory, relating, body and object use, language, and social and self-help, scored by parents or teachers for children over 2 years old. The scale is scored “0” to “158” points, with points over “67” indicating a diagnosis of autism.

Secondary outcomes included the autism diagnostic observation scale, autism diagnostic interview-revised, social responsiveness scale, clinical global impression scale, social interaction questionnaire, Child Behavior Checklist, and Wexner Rating Scale.

### 2.3 Information source and search strategy

**Figure 1:**
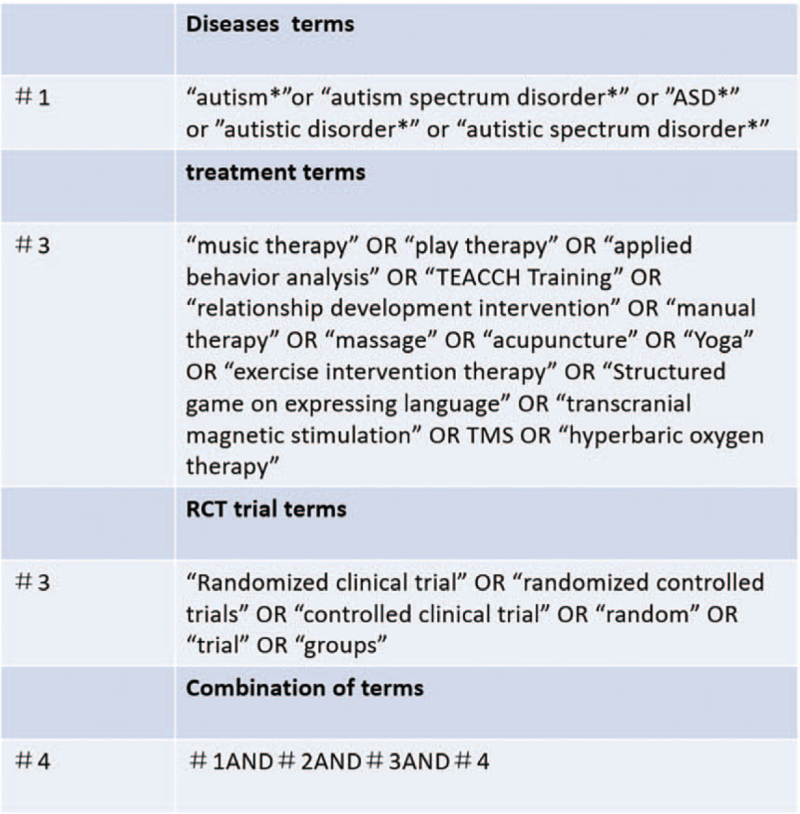
Search strategy in MEDLINE.

Eight electronic databases, including 4 English databases (MEDLINE, Web of Science, EMBASE, and the Cochrane Library) and 4 Chinese databases (China National Knowledge Infrastructure [CNKI], SinoMed, Wanfang Database [WF], and the China Science and Technology Journal Database [VIP]), will be searched for eligible studies published before October 31, 2021, regardless of language and publication type. Different strategies will be applied to electronic databases based on treatment terms (music therapy, play therapy, applied behavior analysis, TEACCH training, relationship development intervention, manual therapy, massage, acupuncture, and yoga), disease terms (autism, ASD, and autistic disorder), and RCT terms (randomized clinical trials, randomized controlled trials, controlled clinical trials, random trials, trials, and groups) (Fig. 1).

### 2.4 Data collection and management

#### 2.4.1 Screening of studies

**Figure 2:**
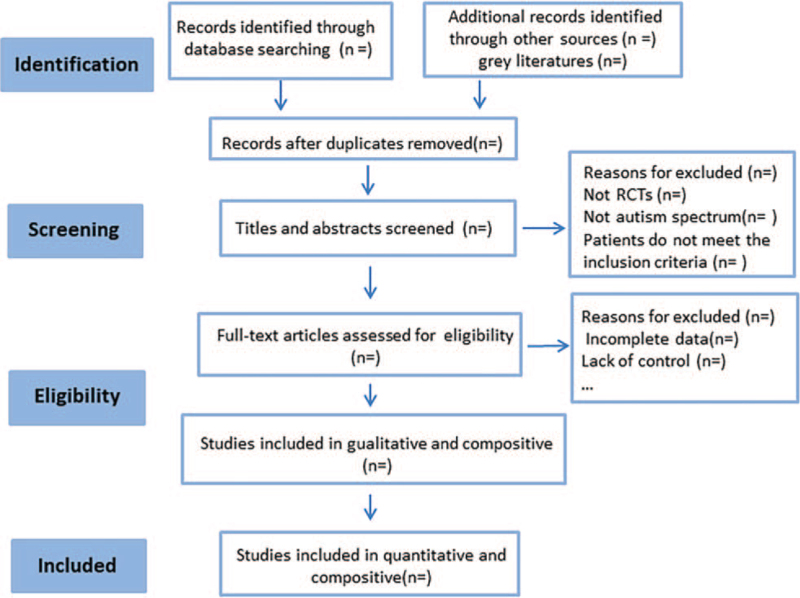
Preferred Reporting Items for Systematic Reviews and Meta-Analysis (PRISMA) flow chart of the study process.

The evaluators were trained centrally, and the documents in the library were screened according to the criteria to ensure screening standardization. Two independent researchers (M. S. and J. X.), both masters of (non)acupuncture and moxibustion, will monitor and evaluate the screening process. Two reviewers screened articles that met the inclusion criteria according to the title and abstract content. After the screening, another reviewer reviewed the articles. Disagreements between the 2 authors will be resolved through consensus or discussion with a third author (W. X.Q.). The systematic evaluation plan and full text will be elaborated upon during the selection. The main process consists of the following steps (Fig. 2): use EndNote to annotate compliant literature and exclude duplicate studies; read the title and abstract of each paper carefully and exclude those that clearly do not meet the inclusion criteria based on their content; and analyze and determine any duplicated publications.

#### 2.4.2 Data extraction

Data were collected by 2 independent researchers (M. S. and J. X.) and verified by a third party, and all disagreements were resolved through consensus. Data related to study identification (author's name, date of publication, type of study, region of publication, and journal/magazine name), study characteristics (study setting, study design, non-pharmaceutical therapy study inclusion criteria, and inclusion criteria for autism spectrum), inclusion criteria (age, onset time, sex, and autism rating scale), intervention type (acupuncture, manual therapy, massage, yoga and exercise, mobilization, and training), and other factors that may introduce errors (funding sources, key conclusions drawn by the authors, evaluation of confounders, and references to other studies) will be included. If the information is clearly incorrect or incomplete, the first author will be contacted via email. Document was deleted when no response was received.

### 2.5 Assessment risk of bias and reporting of study quality

The risk of bias for the included studies will be independently assessed by 2 evaluators using the Cochrane Risk of Bias Tool,^[28]^ and any differences will be resolved through consensus or discussion with a third evaluator. In this assessment, studies were categorized as low bias, high bias, and unclear according to the following 7 factors: random sequence generation (selection bias), allocation concealment (selection bias), blinding of participants and personnel (performance bias), blinding of outcome assessment (detection bias), incomplete outcome data (attrition bias), selective reporting (reporting bias), and other biases.

### 2.6 Data synthesis and statistical analysis

#### 2.6.1 Standard pair-wise meta-analysis

Data analysis and paired meta-analysis will be performed using the RevMan software (version 5.3; Cochrane Collaboration). Relative risk and standardized mean difference were selected as expressions of effect size for dichotomous and continuous variables, with 95% confidence intervals (95% CI). The Q and *I* ^2^ tests were used to detect heterogeneity in the included studies. If *I* ^2^ < 50% and *P* > .10, then the study has no statistical heterogeneity or less heterogeneity, and the fixed effects model can be selected; otherwise, the random effects model can be used.

#### 2.6.2 Network meta-analysis

Owing to the anticipated heterogeneity, NMA within the Bayes framework will be performed with GEMTC 0.14.3 software based on a random effects model of indirect comparison results. The model is calculated using the Markov chain Monte Carlo algorithm.^[29]^ Four chains will be used for the simulation analysis with an annealing time of 20,000 and an iteration time of 50,000. The estimation and inference are carried out under the assumption that the Markov chain Monte Carlo method has reached a stable convergence state. If a closed loop is present, the node analysis method is used to evaluate the inconsistency between direct and indirect evidence. The ranking of the different outcomes for each treatment was summarized by evaluating the area under the cumulative ranking curve. The high surface area of the cumulative ranking curve represents good non-pharmacological interventions for autism.

#### 2.6.3 Assessment of reporting biases

Funnel plots will be used to assess the possibility of a small-sample publication bias when 10 or more studies are included. An asymmetrical, non-inverted funnel shape indicated possible publication bias. This may be related to the small sample size, hidden distribution, and inadequate implementation of the blind method.

#### 2.6.4 Assessment of subgroup and sensitivity

In the case of heterogeneous results, a subgroup analysis will be conducted. In the presence of more than 2 variables, meta-regression was performed. A sensitivity analysis was performed to determine data reliability based on missing data, sample size, and heterogeneity.

#### 2.6.5 Evaluating the evidence quality

Evidence quality will be evaluated by 2 independent authors in accordance with the Grading of Recommendations Assessment Development and Evaluation (GRADE) and classified as follows^[30]^: high, moderate, low, and very low quality. Five factors were considered in GRADE's application of network meta-analysis of evidence quality assessment: risk of bias, indirectness, inconsistency, imprecision, and publication bias.

## 3 Discussion

Autism causes serious damage to the physical and mental health of children owing to the refractory and lifelong nature of its core symptoms. RCTs have shown that non-pharmaceutical therapies can effectively alleviate the core symptoms of autism. Due to the lack of reported side effects, this treatment is easily accepted by patients. However, no comparison has been conducted among the different non-pharmacological interventions applied to alleviate the core symptoms of autism. This systematic review and network meta-analysis will compare the effects of multiple nonpharmacological interventions by combining direct and indirect data. The ranking probability of treatment effects will also be obtained within the Bayesian framework to comprehensively analyze different non-pharmacological interventions. All available evidence regarding the impact of these therapies on the core symptoms of autism will be examined.

### 3.1 Ethics and dissemination

No ethical review was required, because this study did not involve raw data collection. These results provide strong evidence for the treatment of autism using non-pharmacological options. These findings will help clinicians make treatment decisions for autism. This systematic review will be published in a peer-reviewed journal and at an international conference.

### 3.2 Strengths and limitations

This is the first systematic review and network meta-analysis of the efficacy and safety of nonpharmacological therapies for the relief of autism core symptoms. The results will reveal the advantages and disadvantages of various non-pharmaceutical treatment methods for autism and provide a basis for their clinical application to improve the quality of life of patients. Only studies published in English and Chinese were included because of language limitations; this phenomenon may lead to selection bias. In addition, the age and region of the patients and the quality of the literature may increase the possibility of heterogeneity. The methodology and quality assessment will follow systematic evaluation guidelines and standards to minimize the risk of bias.

## Author contributions

**Conceptualization:** Xin Jiang, Weixun Qin

**Data curation:** Jiang Xiao, Xiaoqing Xu.

**Data extraction:** Jiang Xiao, Xiaoqing Xu

**Funding acquisition:** Qing Yuan.

**Investigation:** Song Min, Qing Yuan, Min Song.

**Methodology:** Xin Jiang, Weixun Qin, Song Min

**Project administration:** Qing Yuan

**Resources:** Weixun Qin, Qing Yuan

**Software:** Xin Jiang, Min Song.

**Writing – original draft:** Xin Jiang, Min Song, Weixun Qin

**Writing – review & editing:** Jiang Xiao, Qing Yuan
